# Supplementary material for: Research on China’s Risk of Housing Price Contagion Based on Multilayer Networks
Source: Entropy (Basel). 2022 Sep 15;24(9):1305. doi: 10.3390/e24091305 (PMC9498244; doi:10.3390/e24091305)
Supplement: Supplementary file 1 [file entropy-24-01305-s001.zip › entropy-1815079-supplementary.pdf]

Supplementary Materials: Research on China's Risk of Housing Price Contagion Based on Multilayer Networks

Appendix I. Construction of Multilayer Networks

1. Calculation of Transfer Entropy

Schreiber (2000) proposed TE based on the concept of information entropy, which can describe the nonlinear directional coupling relationship between variables. The following is a definition of TE:

$$TE(X \to Y) = I(X, Y \mid Y_{T+1}) \tag{S1}$$

where X and Y represent the historical state variables of two different time series and Y_{T+1} denotes the future state of the sequence Y. TE is a measure of the actual predictable amount of transfer between variables. The TE of Y to X is defined as the conditional mutual information of current X, historical $X(x^-)$, and historical $Y(y^-)$ expressed as the degree to which the uncertainty of X is reduced when Y is known, indicating the role of Y in predicting X.

To solve the parameter coordination problem of TE, following the steps of Chen et al.(2014), the time-series data are first symbolized, and the information transfer of two time series is then obtained using the TE calculation method.

The calculation method of TE is as follows.

(1) Considering two time series $X: \{x_1, x_2, x_3, \ldots, x_t\}$ and $Y: \{y_1, y_2, y_3, \ldots, y_t\}$, the symbolization method of permutation entropy is used to divide the continuous time series into n non-overlapping intervals, each of which is represented by a different symbol or value.

(2) The original sequences $\{X\}$ and $\{Y\}$ can be converted into the symbolic sequences $\{I, t = 1, 2, \ldots, T\}$ and $\{J, t = 1, 2, \ldots, T\}$, respectively. Namely, the original sequence is divided into different thresholds, and the data are mapped to the symbol sequence space based on the interval in which the data fall.

(3) Sort the reconstructed vectors I_t and J_t, count the occurrences of each symbol to obtain different state transition probabilities, and obtain the information transmission of the sequence $J \to I$ according to the calculation formula of information entropy. The calculation formula is

$$TE_{J \to I} = \sum p(i_{t+1}, i_t^{(k)}, j_t^{(l)}) \log \frac{p(i_{t+1} \mid i_t^{(k)}, j_t^{(l)})}{p(i_{t+1} \mid i_t^{(k)})} \tag{S2}$$

where i_t and j_t denote the corresponding symbol values when X and Y are mapped

to I and J at time t, respectively, and i_{t+1} is the value corresponding to time $t+1$. $p(\bullet)$ is the probability of event occurrence. $p(i_{t+1}, i_t^{(k)}, j_t^{(l)})$, $p(i_{t+1} | i_t^{(k)}, j_t^{(l)})$, and $p(i_{t+1} | i_t^{(k)})$ represent the joint probability density function and the conditional probability density function, respectively. $i_t^{(k)}$ and $j_t^{(l)}$ denote k-order and l-order Markov processes, respectively. Typically, we examine the impact of the uncertainty of the next period of information; therefore, we set $k = l = 1$.

Similarly, we can calculate the TE of the sequence $I \to J$ as follows:

$$TE_{I \to J} = \sum p(j_{t+1}, j_t^{(k)}, i_t^{(l)}) \log \frac{p(j_{t+1} | j_t^{(k)}, i_t^{(l)})}{p(j_{t+1} | j_t^{(k)})} \quad \text{(S3)}$$

From the aforementioned formula, it can be seen that the TE of $J \to I$ is not necessarily equal to that of $I \to J$. Using symbolic TE, we can describe the asymmetric correlation between two sequences and the direction of information transmission.

In real housing price transactions, changes in housing prices in big cities will play a substantial guiding role, while changes in housing prices in some small cities will also affect housing prices in the central metropolis. However, these two strengths are not always equal. Therefore, the asymmetrical measurement method better reflects the asymmetrical relationship between urban housing prices.

To briefly explain how to calculate the TE $TE_{J \to I}$ of $J \to I$, A, B, and C are used instead of i_{t+1}, i_t, and j_t, respectively. For example, if J = [0.4, 0.6, 0.55, 0.6, 0.5, 0.3, 0.4, 0.2, 0.57, 0.64, 0.5] and I = [0.4, 0.6, 0.5, 0.4, 0.7, 0.8, 0.65, 0.46, 0.44, 0.5, 0.2], then A = [0.6, 0.55, 0.6, 0.5, 0.3, 0.4, 0.2, 0.57, 0.64, 0.5], B = [0.4, 0.6, 0.55, 0.6, 0.5, 0.3, 0.4, 0.2, 0.57, 0.64], and C = [0.4, 0.6, 0.5, 0.4, 0.7, 0.8, 0.65, 0.46, 0.44, 0.5]. Therefore, the calculation formula of TE can be rewritten as

$$\begin{aligned} TE_{J \to I} &= \sum p(ABC) \log \frac{p(ABC)p(B)}{p(AB)p(BC)} \\ &= \sum p(ABC) \log p(ABC) + \sum p(B) \log p(B) \\ &\quad - \sum p(AB) \log p(AB) - \sum p(BC) \log p(BC) \end{aligned} \quad \text{(S4)}$$

Based on the TE method, the incidence matrix of housing prices can thus be calculated as follows:

$$\mathbf{TE\_W} = \begin{bmatrix} 0 & TE(1,2) & \cdots & TE(1,n-1) & TE(1,n) \\ TE(2,1) & 0 & \cdots & TE(2,n-1) & TE(2,n) \\ \vdots & \vdots & \ddots & \vdots & \vdots \\ TE(n-1,1) & TE(n-1,2) & \cdots & 0 & TE(n-1,n-1) \\ TE(n,1) & TE(n,2) & \cdots & TE(n,n-1) & 0 \end{bmatrix} \quad \text{(S5)}$$

where the TE $TE(i, j)$ represents the information transfer from region I to region J. The diagonal element is set to 0 because there is no information transmission between housing prices in the same city.

2. Calculation of Generalized Variance Decomposition

The research on the application of GVD to describe the relationship between different units can be traced back to Diebold and Yilmaz (2014), who first used this method to describe the risk relationship between banks. Diebold and Yilmaz found that the row sum of the GVD results is not always 1. Thus, the variance decomposition results can be processed in row standard words to ensure that the sum of each individual decomposition totals 1. Notably, the matrix constructed using the GVD is also asymmetric, and the GVD matrix is also a method for measuring the asymmetric correlation of housing price spillovers across regions. Following Diebold and Yilmaz, the calculation method of the constructed GVD contagion matrix is as follows.

First, for the VAR(p) process of N variables (see Equation (S6)), the form of the moving average can be expressed as follows (see Equation (S7)):

$$x_t = \sum_{i=1}^{p} \phi_i x_{t-i} + \varepsilon_t \tag{S6}$$

$$x_t = \sum_{i=0}^{\infty} A_i \varepsilon_{t-i} \tag{S7}$$

where $\varepsilon \sim (0\ ,\ \Sigma)$ denotes an independent and identically distributed random perturbation vector. The coefficient matrix A_i of the moving average formula of $N \times N$ order is subject to the following recursive process:

$$A_i = \phi_1 A_{i-1} + \phi_2 A_{i-2} + \cdots + \phi_p A_{i-p} \tag{S8}$$

where A_0 denotes a unit matrix of order N, and when $i < 0$, $A_i = 0$.

The variance contribution refers to the proportion $d_{ij}(h)$ of the H-step forecast error variance of y_i that can be explained by y_j when the variable y_i is subject to external shocks. This indicator reflects the degree to which a variable change is influenced by other variables in the system as well as by itself. The proportion of the forecast error is a fundamental component in calculating the housing price spillover association.

The elements of the GVD matrix for the H-step forecast are calculated as follows:

$$d_{ij}^{H} = \frac{\sigma_{ii}^{-1} \sum_{h=0}^{H-1} (e_i' A_h \Sigma e_j)^2}{\sum_{h=0}^{H-1} (e_i' A_h \Sigma A' e_i)} \tag{S9}$$

where e_j is the unit vector whose element at j is 1 and the rest is 0, Σ denotes the covariance matrix of the random disturbance vector ε_t, σ_{ii} is the ε_t standard deviation, H denotes the forecast period, and h is the disturbance in the moving average formula term lag order.

Because the row sum of the GVD is not always 1, the elements in the generalized variance matrix are standardized to better analyze the spillover relationship between housing prices, and the calculation method is as follows:

$$\tilde{d}_{ij} = \frac{d_{ij}}{\sum_{j=1}^{N} d_{ij}} \tag{S10}$$

Subsequently, the variance decomposition matrix can be obtained as

$$D_{ij}(h) = \begin{pmatrix} \tilde{d}_{11} & \tilde{d}_{12} & \cdots & \tilde{d}_{1N} \\ \tilde{d}_{21} & \tilde{d}_{22} & \cdots & \tilde{d}_{2N} \\ \vdots & \vdots & \ddots & \vdots \\ \tilde{d}_{N1} & \tilde{d}_{N2} & \cdots & \tilde{d}_{NN} \end{pmatrix}_{N\times N} \tag{S11}$$

The diagonal of the obtained GVD matrix is set to 0; only the elements related to housing price overflow are retained. The processed variance decomposition matrix is

$$\mathbf{VD\_W} = \begin{bmatrix} 0 & \tilde{d}_{1,2} & \cdots & \tilde{d}_{1,N-1} & \tilde{d}_{1,N} \\ \tilde{d}_{2,1} & 0 & \cdots & \tilde{d}_{2,N-1} & \tilde{d}_{2,N} \\ \vdots & \vdots & \ddots & \vdots & \vdots \\ \tilde{d}_{N-1,1} & \tilde{d}_{N-1,2} & \cdots & 0 & \tilde{d}_{N-1,N} \\ \tilde{d}_{N,1} & \tilde{d}_{N,2} & \cdots & \tilde{d}_{N,N-1} & 0 \end{bmatrix} \tag{S12}$$
